# Agricultural youth injuries: An updated review of cases from U.S. news media reports, 2016–2021

**DOI:** 10.3389/fpubh.2022.1045858

**Published:** 2022-11-18

**Authors:** Bryan P. Weichelt, Serap Gorucu, Rick R. Burke, Marsha A. Salzwedel, Dennis J. Murphy, Barbara C. Lee

**Affiliations:** ^1^National Children's Center for Rural and Agricultural Health and Safety, National Farm Medicine Center, Marshfield Clinic Research Institute, Marshfield, WI, United States; ^2^Department of Agricultural and Biological Engineering, University of Florida, Gainesville, FL, United States; ^3^Department of Agricultural and Biological Engineering, Penn State University, State College, PA, United States

**Keywords:** youth, child, agriculture, injuries, news reports, safety, media, AgInjuryNews

## Abstract

**Introduction:**

Fatal and non-fatal youth (ages 0–17) injuries in U.S. agriculture continue to be a significant public health concern. Despite sustained work and attention from federally supported research programming, we continue to observe an unacceptably high number of life-altering and life-ending traumatic injuries to youth in agricultural environments. Likewise, there is still a gap in stringent systematic agricultural injury and/or illness surveillance at the federal level. This paper will provide an updated review of child agricultural injuries from U.S. news media reports, expanding upon this author team's initial 2018 report.

**Methods:**

Data collection from 2016 to 2021 occurred as part of the AgInjuryNews initiative, and data were coded according to the Farm and Agricultural Injury Classification (FAIC) system and the Occupational Injury and Illness Classification System (OIICS). The AgInjuryNews system primarily contains news media reports. Categorical variables were analyzed and compared using a chi-square test. In addition, the Jonckheere-Terpstra test for trend was used to test the yearly change in the number of youth injuries.

**Results:**

We observed a general decrease in agricultural injuries compared to the original 2015–2017 dataset. Younger children (<5 years-old) and males were more often injured and more fatally injured than older children and females, respectively. Males and older victims were more likely to suffer an occupational-related injury compared to females and younger victims, respectively. Vehicles remained a major source of injuries, with tractors comprising 28%, and ATVs/UTVs comprising 26% of all injuries. Roadway incidents involving tractors and UTVs were less often fatal compared to non-roadway incidents, while ATVs were more fatal on roadways.

**Discussion:**

This updated review shows childhood agricultural injuries and fatalities continue to be a major public health concern within the US. It is unclear if the trend downward in injuries is due to reporting, data capture methods, or a true decrease in injuries. These data continue to be of interest to stakeholders in academia, public health, government, and private industry—user groups who regularly and consistently seek this type of information, often from multiple data sources, including as registered users on AgInjuryNews.org. These data identify emerging issues within the industry and further inform national and international planning committees' work.

## Introduction

Agriculture remains one of the most dangerous occupations in the US and is the only occupation where children are legally allowed and often expected to actively engage on the worksite (1, 2). As of 2014, an estimated 893,000 youth under 20 years of age live on farms, 454,000 of whom perform work on these farms, and an additional 266,000 are hired laborers under the age of 20 (1). Upwards of 25 million youth visit farms each year, 95% of whom are frequent or repeat visitors (3). Despite an abundance of materials and resources for safeguarding youth in and around agriculture, young workers are nearly eight times as likely to be fatally injured in agriculture compared to all other industries combined, with 48% of all youth occupational fatalities occurring in agriculture (4, 5).

Despite this, there is currently no central data repository for youth agricultural injuries or fatalities in the U.S. Even within the most respected information sources of occupational fatalities, the Bureau of Labor Statistics' (BLS) Census of Fatal Occupational Injuries and Illness (CFOI), gaps exist—particularly for working and non-working youth in agriculture (6). These gaps are well-described in the current literature, with a potential 88% of non-fatal agriculture-related injuries escaping traditional surveillance methods (7).

In a recent study of U.S. emergency department admissions, the authors uncovered 62,079 people treated for agricultural related injuries between 2015 and 2019, of which 30% of those injured were youth (age 0–17) (8). Approximately 10 children presented to U.S. emergency rooms with agricultural injuries every day during that study period (8). Furthermore, a child dies every 3 days in an agriculture-related incident, nearly half of which involve transportation (9).

The key aim of this paper is to provide an updated review of youth (age 0–17) agricultural injuries by analyzing data harvested from U.S. news media reports. Our previous review, published in 2018, was an initial exploratory look at the first national dataset of its kind (10). News media reports have shown to be a powerful and useful tool in supplementing existing data sets and providing surveillance to previously uncaptured injuries.

Given the ever-evolving field of production agriculture and limited availability of national agricultural injury surveillance data, an updated review is presumably welcomed, even necessary, for agricultural safety and health stakeholders in academia, government, and private industry—user groups who regularly and consistently seek this type of information, as evidenced by a recent report in safety (11).

## Materials and methods

### Data collection

Data were collected as part of AgInjuryNews operations; detailed collection methods are further described in separate papers (10, 12). Contributions to these data are regularly supplemented through formal partnerships with several of the U.S. NIOSH-funded Agricultural Safety and Health Research Centers, the Canadian Agricultural Safety Association, and informal partnerships with agricultural safety and health community leaders and stakeholders.

### Coding of data

Data were coded according to the Farm and Agricultural Injury Classification (FAIC) system and the Occupational Injury and Illness Classification System (OIICS) (13, 14). The injury reports were collected, and coded by a primary data specialist. All cases were then coded (FAIC, OIICS, and AgInjuryNews codes) by a second coder, with agreement analyses suggesting “almost perfect” agreement (κ = 0.85, κ_α_ = 0.82) (13). Finally, at least 10% of all injury reports were randomly selected to be reviewed and coded by a third coder for additional quality control. Coder reliability analysis showed a strong interrater reliability (13). Incident location is coded at the highest resolution available with a county-level minimum and geocoded using Google Maps Geocoding API (15).

### Analyses

Descriptive and summary statistics were determined for all cases in AgInjuryNews from 2016 to 2021. Geographic distributions were mapped using Tableau. We compared demographics, incident year, and FAIC and OIICS codes across fatal and non-fatal injuries, occupational vs. non-occupational, tractor, and ATV/UTV injury cases, mirroring the initial review. Categorical variables were analyzed and compared using a chi-square test. In addition, the Jonckheere-Terpstra test for trend was used to test the yearly change in the number of youth injuries. Statistical analyses were conducted in IBM SPSS Statistics version 27.0.

## Results

**Figure 1:**
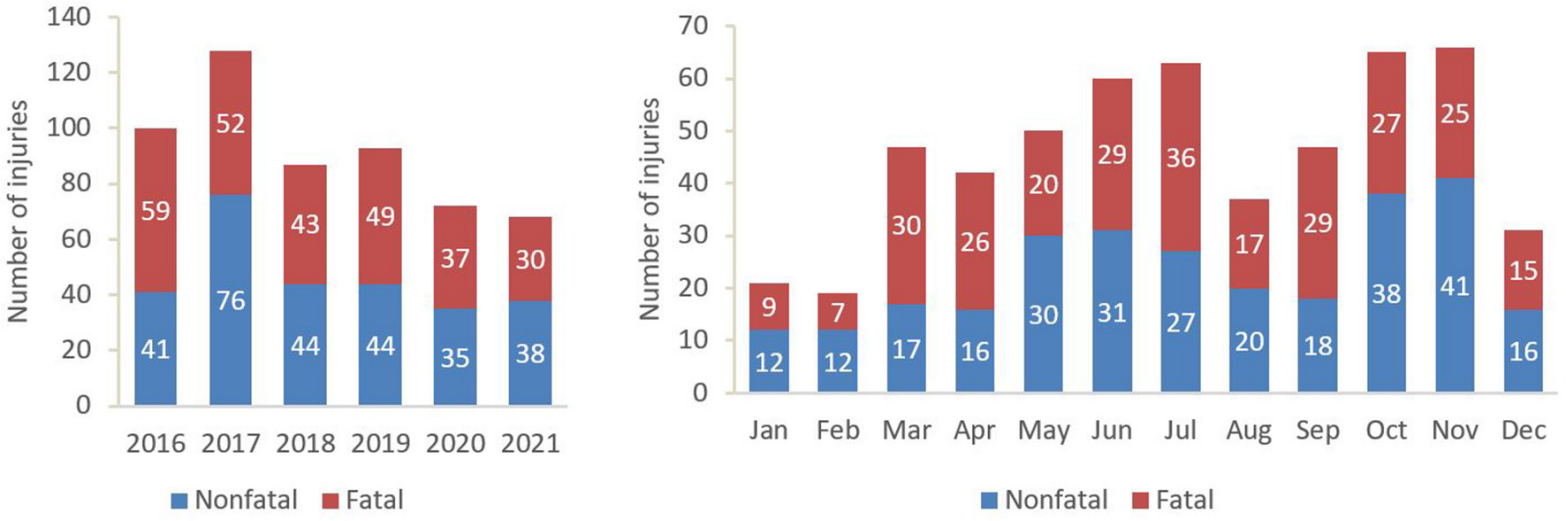
Yearly and monthly distribution of agricultural youth injuries.

The study identified a total of 548 agricultural youth injuries (270 fatal and 278 non-fatal) between 2016 and 2021. Figure 1 summarizes the yearly and monthly distribution of youth injuries. The overall number of injuries decreased over time (p for trend 0.039 using Jonckheere-Terpstra) and peaked in 2017 with 128 cases. Most injuries occurred in November (*n* = 66) followed by October (*n* = 65) and July (*n* = 63; Figure 1).

**Figure 2:**
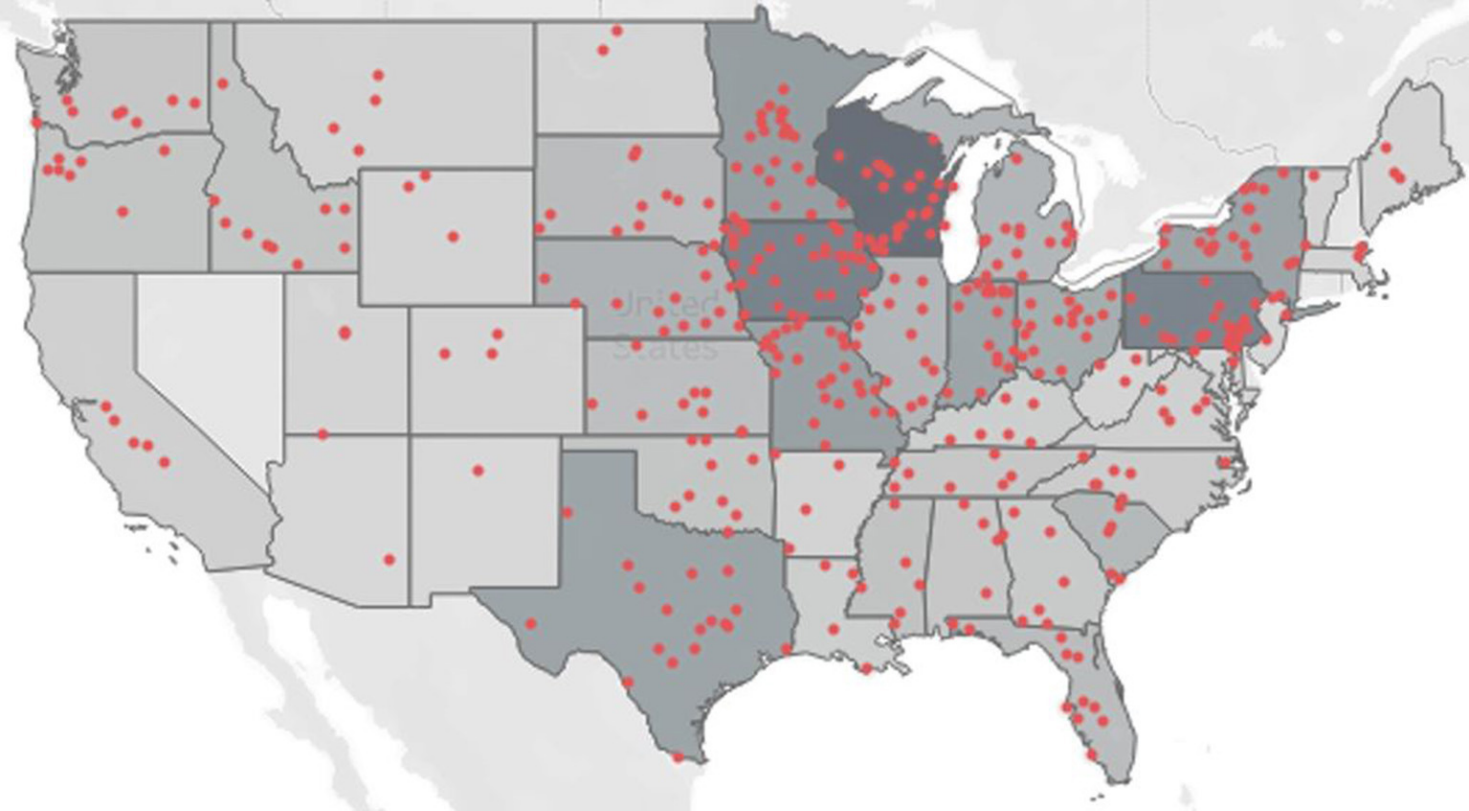
Location of agricultural youth injuries, 2016–2021.

Agricultural youth injury cases occurred in 43 states (Figure 2) and the top three highest numbers of injuries occurred in Wisconsin (*n* = 53), Iowa (*n* = 43), and Pennsylvania (*n* = 42).

Almost one-third of the victims were under 5 years old (*n* = 132, 28.8%). The <5 age group had proportionally higher fatal injuries than the other age groups (χ^2^ = 8.258, df = 3, *p* = 0.041). A total of 327 victims were male (73.6%), and the proportion of fatal injuries to male victims was significantly higher than fatal injuries to females (*P* < 0.001).

Occupational work-relatedness was determined by the FAIC codes. In 118 cases, FAIC coding was not possible because of a lack of information. Of those where work status was determinable, most injuries to youth were non-occupational (80.5%). There was no significant association between occupational relatedness and injury outcome (fatal or non-fatal; *p* > 0.05; Table 1). Agricultural injuries to youth mostly involved vehicles (73.6%) and transportation incidents (76.8%).

**Table 1:** Characteristics of injured victims, FAIC and OIICS codes, and injury severity between January 1, 2016 to December 31, 2021.

**Characteristics**	**Total**	**Fatal**	**Non-fatal**	**χ^2^**
	**(*n* = 548)**	**(*n* = 270)**	**(*n* = 278)**	
**Age (valid** ***n*** **=** **459)**				
0–4 years	132 (28.8%)	88 (66.7%)	44 (33.3%)	χ^2^ = 8.258, df = 3, *p* = 0.041
5–9 years	120 (26.1%)	63 (52.5%)	57 (47.5%)	
10–14 years	116 (25.3%)	64 (55.2%)	52 (44.8%)	
15–17 years	91 (19.8%)	45 (49.5%)	46 (50.5%)	
Missing age^*^ (*n* = 89, 10 fatal, 79 non-fatal)				
**Gender (valid** ***n*** **=** **444)**				
Male	327 (73.6%)	202 (61.8%)	125 (38.2%)	χ^2^ = 8.611, df = 1, *p* = 0.003
Female	117 (26.4%)	54 (46.2%)	63 (53.8%)	
Missing gender^*^ (*n* = 104, 14 fatal, 90 non-fatal)				
**FAIC code (valid** ***n*** **=** **431)**				
Occupational	81 (18.9%)	44 (54.3%)	37 (45.7%)	χ^2^ = 0.369, df = 1, *p* = 0.543
Non-occupational	348 (81.1%)	176 (50.6%)	172 (49.4%)	
Undeterminable FAIC^*^ (*n* = 118, 49 fatal, 69 non-fatal)				
**OIICS-injury source (valid** ***n*** **=** **534)**				
Vehicles	392 (73.4%)	181 (46.1%)	211 (53.8%)	χ^2^ = 15.922, df = 4, *p* = 0.003
Machinery	63 (11.8%)	41 (65.1%)	22 (34.9%)	
Structures	32 (6.0%)	21 (65.6%)	11 (34.4%)	
Persons, plants, animals	27 (5.1%)	9 (33.3%)	18 (66.7%)	
Others	20 (3.7%)	13 (65.0%)	7 (35.0%)	
Unclassifiable Injury source^*^ (*n* = 14, 5 fatal, 9 non-fatal)				
**OIICS-injury event type (valid** ***n*** **=** **527)**				
Transportation	404 (76.8%)	194 (48.0%)	210 (52.0%)	χ^2^ = 5.743, df = 4, p = 0.219
Contact with objects and equipment	70 (13.3%)	34 (48.6%)	36 (51.4%)	
Exposure to harmful substances	25 (4.8%)	18 (72.0%)	7 (28.0%)	
Violence and injuries by persons or animals	12 (2.3%)	5 (41.7%)	7 (58.3%)	
Others	15 (2.9%)	7 (46.7%)	8 (53.3%)	
Unclassifiable injury event type^*^ (*n* = 22, 10 fatal, 12 non-fatal)				

FAIC, Farm and Agricultural Injury Classification; OIICS, Occupational Injury and Illness Classification System.

* Missing and undeterminable/unclassifiable cases not included in the percentages or in chi-square analysis.

Column percentages are shown for “total.” Row percentages are shown for fatal/non-fatal.

Characteristics of occupational and non-occupational injuries are given in Table 2. Most of the occupational-related fatalities and injuries were associated with the occupation of farming (FAIC-1, *n* = 79). Non-occupational injuries mostly occurred as a result of exposure to farm equipment, tools, and product hazards (FAIC-6, *n* = 216). A total of 95 youth occupants of other vehicles were injured in agricultural roadway incidents (FAIC-9). Other injuries were related to farm structures and landscapes (FAIC-7, *n* = 31) and non-occupational animal-related injuries (FAIC-8, *n* = 6). Males and the 15–17-year age group had higher proportions of occupational injuries.

**Table 2:** Characteristics of occupational and non-occupational injuries.

**Variables**	Undeterminable^*^	**Occupational**	**Non-occupational**	**χ^2^**
**Year**				
2016	18	17 (20.7%)	65 (79.3%)	χ^2^ = 8.269, df = 5, *p* = 0.142
2017	19	14 (12.8%)	95 (87.2%)	
2018	10	18 (23.4%)	59 (76.6%)	
2019	24	17 (24.6%)	52 (75.4%)	
2020	23	11 (22.4%)	38 (77.6%)	
2021	25	4 (9.3%)	39 (90.7%)	
**Gender**				
Male	23	70 (26.7%)	192 (73.3%)	χ^2^ = 17.036, df = 1, *p* < 0.0001
Female	65	6 (6.4%)	88 (93.6%)	
**Age categories**				
0–4	11	2 (1.7%)	119 (98.3%)	χ^2^ = 67.050, df = 3, *p* < 0.0001
5–9	22	12 (12.2%)	86 (87.8%)	
10–14	34	24 (29.3%)	58 (70.7%)	
15–17	23	32 (47.1%)	36 (52.9%)	

* Undeterminable variables not included in chi-square analysis.

Tractor-related incidents were associated with 153 injuries, and 77 of these happened on roadways (Table 3). Similar to the general results most of the victims in tractor-related incidents were males and in the 0–4 age group. In terms of the victims' roles, most of the victims were tractor passengers (39.6%). We identified 46 other vehicle victims injured as a result of tractor-related incidents on roadways. This included youth passengers (*n* = 31) and youth drivers (*n* = 15).

**Table 3:** Characteristics of tractor-related injuries.

**Variables**	**Tractor**		
	**Total** **(*n* = 153)**	**Fatal** **(*n* = 61)**	**Non-fatal** **(*n* = 92)**
**Year**	χ^2^ = 8.251, df = 5, *p* = 0.143		
2016	29 (19.0%)	12	17
2017	35 (22.9%)	10	25
2018	23 (15.0%)	14	9
2019	20 (13.1%)	6	14
2020	27 (17.6%)	13	14
2021	19 (12.4%)	6	13
Age^*^	χ^2^ = 5.717, df = 3, *p* = 0.126		
0–4 years	42 (33.3%)	24	18
5–9 years	31 (24.6%)	10	21
10–14 years	21 (16.7%)	11	10
15–17 years	32 (25.4%)	12	20
Gender^*^	χ^2^ = 0.071, df = 1, *p* = 0.789		
Male	79 (73.8%)	39	40
Female	28 (26.2%)	13	15
Events^*^	χ^2^ = 10.569, df = 2, *p* = 0.005		
Roadway	77 (52.7%)	21	56
Non-roadway	60 (41.1%)	32	28
Contact	9 (6.2%)	5	4
Role^*^	χ^2^ = 8.437, df = 3, *p* = 0.038		
Tractor passenger	55 (39.6%)	20	35
Other vehicle occupants	46 (33.1%)	14	32
Bystanders	23 (16.5%)	15	8
Tractor operator	15 (10.8%)	5	10

* Undeterminable/unknown variables not shown and they are not included in chi-square analysis.

A total of 143 victims sustained injuries *via* ATV/UTV-related incidents, and more than half of these injuries were fatal (*n* = 73, 51%). Table 4 shows the injury characteristics of these incidents. Most of ATV/UTV injuries occurred as non-roadway incidents and more than half of the victims were youth operators (Table 4).

**Table 4:** Characteristics of ATV/UTV related injuries.

**Variables**	**ATV (*****n*** = **98)**			**UTV (*****n*** = **45)**			**Total**
	**Total**	Fatal^*^ **(*n* = 55)**	**Non-fatal** ** (*n* = 43)**	**Total**	**Fatal** ** (*n* = 18)**	**Non-fatal** ** (*n* = 27)**	
**Year**	χ^2^ = 7.863, df = 5, *p* = 0.164			χ^2^ = 4.583, df = 5, *p* = 0.469			
2016	22	17	5	4	1	3	26 (18.2%)
2017	26	10	16	16	4	12	42 (29.4%)
2018	15	8	7	5	2	3	20 (14.0%)
2019	10	6	4	6	3	3	16 (11.2%)
2020	11	7	4	10	5	5	21 (14.7%)
2021	14	7	7	4	3	1	18 (12.6%)
Age^*^	χ^2^ = 1.684, df = 3, *p* = 0.640			χ^2^ = 1.287, df = 3, *p* = 0.732			
0–4 years	9	4	5	4	1	3	13 (10.2%)
5–9 years	27	18	9	7	3	4	34 (26.6%)
10–14 years	40	22	18	17	9	8	57 (44.5%)
15–17 years	15	9	6	9	5	4	24 (18.8%)
Gender^*^	χ^2^ = 1.074, df = 1, *p* = 0.300			χ^2^ = 3.922, df = 1, *p* = 0.048			
Male	63	39	24	22	13	9	85 (65.9%)
Female	26	13	13	18	5	13	44 (34.1%)
Events^*^	χ^2^ = 1.119, df = 1, *p* = 0.290			χ^2^ = 4.132, df = 1, *p* = 0.042			
Non-roadway	84	45	39	37	16	21	121 (86.4%)
Roadway	13	9	4	6	-	6	19 (13.6%)
Role^*^	χ^2^ = 8.950, df = 1, *p* = 0.003			χ^2^ = 2.730, df = 1, *p* = 0.098			
Operator	51	34	17	14	7	7	65 (52.8%)
Passenger	33	11	22	25	6	19	58 (47.2%)

* Undeterminable/unknown variables not shown and they are not included in chi-square analysis.

## Discussion

This study of U.S. news media reports expands upon current known trends from 2015 to 2017, providing further insight into how the landscape of child agriculture injuries is changing. We continue to observe an overrepresentation of male injuries across all variables, notably seeing an increase in the proportion of males suffering occupational injuries vs. non-occupational injuries compared to females. Adult supervision is one known strategy for reducing injuries, however, some studies have found that supervision alone is not always effective in the agricultural environment, especially for young children, who lack the developmental abilities to remain safe in this environment (16, 17). Given the young age and non-occupational status of victims [81% of injuries occurred while youth were not working (Table 2)], a better strategy to prevent injuries and fatalities is the removal of young children from work areas and keeping young children away from farm vehicles and machinery.

Transportation continues to be the largest source of injuries to youth on and around farms. While tractors remain a significant injury agent, ATV/UTV injuries appear to trend downward over time. The cause for this is difficult to identify, as we have seen regulations surrounding these vehicles change on a county by county basis throughout several states (18, 19). This may also be due to a limitation in news agencies' tendencies to report on ATV/UTV injuries and incidents, as well as underreporting or reductions in seeking medical care. More in-depth analyses on the impact of ATV/UTV regulations and legislation on fatal and non-fatal injuries would help inform current and future policies.

As mentioned, tractors remain a consistent injury agent in these incidents, including youth operators, passengers, or bystanders. Over 50% of youth tractor injuries occurred to children under 10 years-old; however, age did not appear to have a significant impact on the fatality of an injury. Roadway incidents continued to be more fatal than non-roadway incidents, often involving non-farming victims in passenger vehicles. This emphasizes the need for tractor safety courses aimed at youth operators, as well as bystander and non-working youth safety in areas where tractors are operating. Some U.S. states require youth to be at least 16 years-of-age before operating a tractor; however, some states allow children as young as 12 to be certified for public roadway operations, and there are many exceptions for those remaining on privately-owned land, non-work related activities, and more (20, 21).

Overall, we observed a slight downward trend in injuries reported through news agencies and captured by the AgInjuryNews.org system. This is an excellent finding from this updated review, and suggests that injury prevention efforts with agricultural youth are having a positive impact. However, there may be other explanations for the downward trend. It is possible that some news media are shifting away from reporting on agricultural injuries, or instead focusing more on fewer, more serious cases. There is also the ongoing consolidation of farms across the U.S. and more corporate operations buy-out of smaller farms. These larger operations can be less willing to employ young workers, particularly young children under 14 that may have otherwise performed work duties on a family-owned operation. The most recent, comprehensive data on the number of youth agricultural workers is from 2014, making this determination difficult.

This downward trend could also be due to changes in the manner of reporting that is not being captured by the current search methods of AgInjuryNews.org. While the system utilizes many avenues of data collection with comprehensive search terms, significant changes in the wording and content of news reports can result in a lower capture rate. Finally, it is unknown what effect the COVID-19 pandemic had on agricultural hazard and risk exposures involving youth. Some studies suggest that youth did spend more time in the workplace, particularly in 2020 (22). Still, our data herein, along with preliminary death certificate data analyses from the 2020 WI Farm Fatality report development, do not show an increase in the number of injuries or fatalities within that time frame (23).

Though our team reports a decrease of youth injuries in recent years, we cannot claim with any certainty that: (1) this represents actual counts of traumatic agricultural injury in the U.S. or (2) this decline will be sustained. While news media remain one of the few supplemental data sources for non-fatal agricultural injuries, continued monitoring of cases and, preferably, a more rigorous, centralized child agricultural injury surveillance program would be ideal. Expanding and simplifying injury reporting for small and non-employing operations can further improve capture rates of agricultural injuries.

## Conclusion

We observed a slight downward trend in agricultural youth injuries reported through news media from 2016 to 2021, though we recommend continual monitoring of injuries therein. We also suggest further research and policymaker attention on public roadway safety (e.g., agricultural equipment lighting and marking), tractor operations and limiting extra riders, ATV/UTV usage and regulation, and an emphasis of removing young children from the worksite. Each of these priority areas should be of concern for youth injury prevention.

Media monitoring remains a powerful tool for agricultural injury surveillance, particularly fatalities, and youth cases. Digital reports, such as news media, obituaries, and social media posts, are valuable data sources in an industry lacking a comprehensive injury surveillance system. The AgInjuryNews.org system continues to provide a hypothesis-generating information and a strong foundation for collaborations and partnerships throughout agricultural health and safety, including international expansion.

The variety and vastness of our nation's farms and ranches pose serious challenges, with limited formal safety regulations, incentives, or enforcement. However, that should not dissuade us from doing what we can—taking steps toward creating a safer, more sustainable industry and workforce. Continued improvement of our surveillance and injury monitoring methods are essential to ensure accurate and actionable agricultural injury data, to better inform health and safety guidelines, resources, and public policy.

## Data availability statement

The original contributions presented in the study are included in the article/supplementary material, further inquiries can be directed to the corresponding author.

## Author contributions

All authors participated in the conception or design of the work, the acquisition, analysis, or interpretation of data for the work, drafting the work and revising it critically for important intellectual content, final approval of the version to be submitted/published, and all agree to be accountable for all aspects of the work in ensuring that questions related to the accuracy or integrity of any part of the work are appropriately investigated and resolved.

## Funding

Funding support for this work was provided through the National Farm Medicine Center and Marshfield Clinic Research Institute and the USDA National Institute of Food and Agriculture: Project FLA-ABE-006052, Accession number 1025545. Funding for the AgInjuryNews program has been made possible, in part by the National Children's Center for Rural and Agricultural Health and Safety through the National Institute for Occupational Safety and Health Cooperative Agreement U54 OH009568, a cooperative agreement award for the Centers for Disease Control and Prevention/National Institute for Occupational Safety and Health. Funding support was also provided through a safety grant from the Agricultural Safety and Health Council of America (ASHCA), the University of Wisconsin Clinical and Translational Science Award (CTSA) program, through the NIH National Center for Advancing Translational Sciences (NCATS), grant UL1TR002373, and through collaborators, donors, and friends of the National Farm Medicine Center, part of the Marshfield Clinic Research Institute.

## Conflict of interest

The authors declare that the research was conducted in the absence of any commercial or financial relationships that could be construed as a potential conflict of interest.

## Publisher's note

All claims expressed in this article are solely those of the authors and do not necessarily represent those of their affiliated organizations, or those of the publisher, the editors and the reviewers. Any product that may be evaluated in this article, or claim that may be made by its manufacturer, is not guaranteed or endorsed by the publisher.
